# Application of insect-proof nets in pesticide-free rice creates an altered microclimate and differential agronomic performance

**DOI:** 10.7717/peerj.6135

**Published:** 2018-12-21

**Authors:** Guoying Yang, Zhi Guo, Hongting Ji, Jing Sheng, Liugen Chen, Yanwen Zhao

**Affiliations:** 1 College of Resources and Environmental Sciences, Nanjing Agricultural University, Nanjing, Jiangsu, China; 2 Circular Agriculture Research Center, Jiangsu Academy of Agricultural Sciences, Nanjing, Jiangsu, China; 3 Nanjing Institute of Agricultural Sciences in Jiangsu Hilly Area, Jiangsu Academy of Agricultural Sciences, Nanjing, Jiangsu, China

**Keywords:** Insect-proof nets, Microclimate, Agronomic performance, Rice, Plant-environment interactions

## Abstract

**Background:**

Insect-proof nets are commonly used in crop production and scientific research because of their environmental, economic, and agronomic benefits. However, insect-proof nets can unintentionally alter the microclimate inside the screenhouse and therefore greatly affect plant growth and yield. To examine the microclimate and agronomic performance of pesticide-free rice under insect-proof nets, two-year field experiments were carried out in 2011 and 2012.

**Methods:**

In the present study, the experiment was conducted by using a split-plot design considering the cultivation environment (open field cultivation (OFC) and insect-proof nets cultivation (IPNC)) as the main plot and the varieties as the subplot (Suxiangjing3 and Nanjing44).

**Results:**

IPNC significantly reduced the air speed and solar radiation, and slightly increased the daytime soil temperature, daytime air temperature, and nighttime relative humidity. By contrast, the nighttime soil temperature, nighttime air temperature, and daytime relative humidity were relatively unaffected. The grain yield of both rice cultivars decreased significantly under IPNC, which was largely attributed to the reduced panicle number. The reduced panicle number was largely associated with the decreased maximum tiller number, which was positively correlated with the tillering rate, time of tillering onset, and tillering cessation for both rice cultivars under IPNC. In addition, dry matter accumulation significantly decreased for both rice cultivars under IPNC, which was mainly caused by the decreased leaf area duration resulting from the reduced leaf area index. By contrast, the mean net assimilation rate was relatively unaffected by IPNC.

**Discussion:**

Insect-proof nets altered the microclimate in comparison with OFC by reducing the air speed and changing the radiation regime, which significantly affected dry matter production and yield of both japonica rice cultivars. Our results indicated that cultivation measures that could increase the tillering rate and the maximum tiller number under IPNC would lead to a significant increase in panicle number, ultimately increasing grain yield. In addition, maintaining a high leaf area duration by increasing the leaf area index would be important to compensate for the dry matter accumulation losses under IPNC. These findings are critical to provide a theoretical basis for improving agronomic performance of pesticide-free rice under IPNC.

## Introduction

Insect-proof nets provide an ecological and effective approach to control the infection and transfer of plant diseases and insect pests (Guo et al., 2015). They reduce the amount of chemical pesticides application, the health risks for workers, and potential environmental pollution (Möller et al., 2005; Castellano et al., 2008). In addition, insect-proof nets are efficient tools to mitigate the negative effect of harsh weather, such as hail, strong wind, and excessive radiation load (Mahmood et al., 2018; Mupambi et al., 2018). Currently, protected cultivation using insect-proof nets is a common practice in many countries of worldwide (Muleke et al., 2012; Kitta et al., 2014; Vidogbéna et al., 2015).

In China, insect-proof nets are commonly used by farmers in more than 25 provinces to reduce diseases and insect pests and modify the microclimate leading to improved vegetable production and quality in summer and autumn (Xing et al., 2007; Xu, Li & Cao, 2010; Huang et al., 2013). In recent years, many studies have reported that insect-proof nets have significant positive effects on rice production (Lu et al., 2006; Lu et al., 2008; Li et al., 2012; Wu, 2016). They have attracted significant attention and have been applied in pesticide-free rice production by agricultural workers in China. Insect-proof nets applied in rice nurseries could significantly improve seedling quality and reduce pests and diseases, with no chemical pesticides input (Lu et al., 2006; Li et al., 2012). Insect-proof nets applied in rice nurseries could also enhance the resistance of rice plants to rice phanthoppers and southern rice black-streaked dwarf virus during the growing period in the field, which increased grain yield by 8.3–13.4% compared with that of the uncovered control (Hei et al., 2013). Furthermore, insect-proof nets applied during the whole growth period of rice could improve grain quality because of the more favorable microclimate inside the screenhouse compared with ambient conditions (Guo et al., 2015). More importantly, the use of insect-proof nets in rice fields is an optional mode in response to the pressure of reducing greenhouse gas emissions from rice fields. Xu et al. (2017) found that screenhouse cultivation could decrease CH_4_ and N_2_O emissions by 6.6–18.7% and 2.5–21.4%, respectively, and the global warming potential by 6.5–18.7% compared with those in open field conditions. From a research standpoint, insect-proof nets are commonly used in agricultural and ecological studies to examine plant-insect interactions in field conditions while maintaining control over the insect populations (Catangui, Beckendorf & Riedell, 2009; Perillo et al., 2015). In addition, insect-proof nets have been applied in seed breeding or seed production to improve the seed purity of cereal crops by isolating heterologous pollen (Ma et al., 2000; Ma et al., 2002; Wang et al., 2016; Long et al., 2017).

Although insect-proof nets are effective tools for sustainable agricultural production, net covering unintentionally alters the microclimate inside the screenhouse, mainly by decreasing the air speed and changing radiation regime, which markedly affects crop physiology, growth, and yield (Perillo et al., 2015). Insect-proof nets decrease the air speed inside the screenhouse because of the increase in airflow resistance (Tanny, 2013), which could reduce the supply of CO_2_ for photosynthesis (Liu et al., 2017) and increase the temperature and humidity (Harmanto Tantau & Salokhe, 2006; Tanny, 2013). Moreover, insect-proof nets decreased the level of solar radiation and modified the light quality parameters, which greatly affected dry matter production and yield (Raveh et al., 2003; Kittas et al., 2012; Kitta et al., 2014; Liu et al., 2017).

The use of insect-proof nets, whether for crop production or research purposes, requires adequate crop performance within the nets (Lawson et al., 1994). Many studies have investigated the consequential effects of a modified microclimate inside the screenhouse on crop physiology, growth, and yield, for example in tomato (Kittas et al., 2012), sweet pepper (Kitta et al., 2014), soybean (Perillo et al., 2015), and rice (Liu et al., 2017). Most of the previous studies placed emphasis on the impact of the changing radiation regime inside the screenhouse on crop production and yield (Raveh et al., 2003; Kittas et al., 2012; Kitta et al., 2014; Perillo et al., 2015). However, their results were inconsistent in some respects. Kitta et al. (2014) reported that net-covering modified the radiation regime such that the low solar radiation might be more than compensated for by an increase in the fraction of diffuse radiation, which led to a significant increase in the leaf area index and canopy light interception, thereby increasing canopy light use efficiency and crop yield. Perillo et al. (2015) reported that net covering significantly decreased the leaf area index of soybean, possibly caused by low solar radiation inside the screenhouse. The net covering-induced changes in the radiation regime resulted in shaded leaves in the canopy being exposed to a higher illumination level than those in the open field conditions, which significantly increased the total plant biomass. However, Liu et al. (2017) reported that insect-proof nets decreased the photosynthetic rate of a single leaf in the top canopy (*Pn*) because of the decreased solar radiation reaching the rice plants, which significantly decreased the dry matter accumulation and translocation, as well as grain yield. It should be noted that the canopy net assimilation rate was considered to be more accurate than *Pn* in determining constraints on crop production, especially under low solar radiation conditions caused by net covering (Mu et al., 2010). The decrease in the net assimilation rate under low solar radiation at the canopy level was less than that at the single leaf level causing by certain compensation effects (Mu et al., 2010). However, there is less evidence on the effects of net covering on the canopy net assimilation rate at the crop level, and whether these effects are the dominant factors causing the decreased dry matter production. In addition, Liu et al. (2017) found that insect-proof nets significantly decreased the effective panicle numbers because of lower solar radiation levels inside the screenhouse. Tiller production and survival determine the final panicle number, and light intensity and quality are the important factors that affect tillering production and survival (Evers, Vos & Andrieu, 2006; Sparkes, Holme & Gaju, 2006). Previous studies reported that lower solar radiation levels and a lower red/far-red ratio resulted in a slower tillering rate and less effective tillers and therefore, lower panicle numbers, in wheat (Casal, 1988; Xie, Mayes & Sparkes, 2015). Insect-proof nets decreased solar radiation levels and the red/far-red ratio inside the screenhouse (Kitta et al., 2014), which might have a negative effect on tillering production and survival of rice. However, studies on the effects on tillering production and survival of rice under insect-proof nets cultivation are limited. Identifying the effects of insect-proof nets cultivation on different rice varieties would provide important information to aid the development of management strategies to improve agronomic performance of rice grown inside the screenhouse. Genotypic variation for grain quality of japonica rice cultivars in response to the modified microclimate inside the screenhouse has been investigated (Guo et al., 2015). However, a comparison of the effects of net covering on dry matter production and yield in different japonica rice cultivars has rarely been reported.

Thus, in the present study, two-year field experiments were conducted to investigate the effects of insect-proof net cultivation on the microclimate inside the screenhouse and its effects on dry matter production and grain yield of pesticide-free rice. The main objectives of this study were: (1) to identify the effects of insect-proof nets on the microclimate; (2) to determine how a changed microclimate inside the screenhouse affected the leaf area index, dry matter production, and yield of different pesticide-free rice cultivars; and (3) to investigate the effects of insect-proof nets on leaf area duration and the net assimilation rate, and their relative contribution to dry matter production under insect-proof nets.

## Materials and Methods

### Experimental site

Field experiments were conducted in paddy fields in 2011 and 2012 in the Baima Experimental Station of Plant Science of Jiangsu Academy of Agricultural Science, Nanjing, Jiangsu Province, China (31°36′E, 119°11′E). The soil was yellow brown soil with an organic matter content, total nitrogen content, total phosphorus content, available nitrogen content, available phosphorus content and available potassium content of 16.62 g kg^−1^, 0.87 g kg^−1^, 0.24 g kg^−1^, 35.16 mg kg^−1^, 11.84 mg kg^−1^, and 89.23 mg kg^−1^, respectively.

### Experimental design

**Figure 1:**
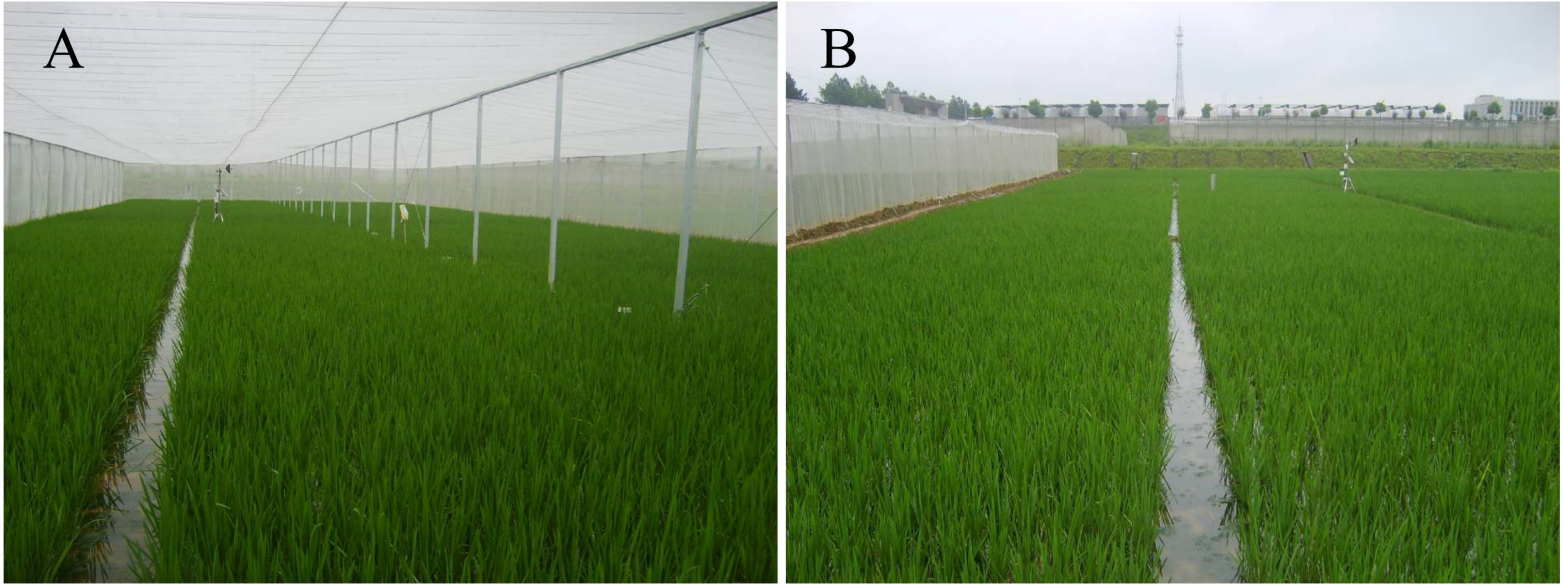
Photograph of insect-proof nets cultivation (A) and open field cultivation (B).

The experiment was conducted by using a split-plot design considering the cultivation environment (open field cultivation (OFC) and insect-proof nets cultivation (IPNC)) as the main plot and the rice variety as a subplot. The experiment was performed in three replicates with a subplot area of 150 m^2^. The experimental cultivars were Suxiangjing3 (SXJ3, medium-maturing medium japonica cultivar) and Nanjing44 (NJ44, early-maturing late japonica cultivar), which were sown on May 16, 2011 and May 18, 2012, respectively. The seedlings were manually transplanted into the experimental plot at a density of two seedlings per hill on June 22, 2011 and June 19, 2012, with a hill spacing of 30 cm ×13 cm, respectively. The insect-proof screenhouse was applied from the transplanting stage to maturity. The external dimensions of the insect-proof screenhouse (white net with 30-mesh) used in this study were 60 m × 20 m × 3 m (length × width × height), in which the spacing of the vertical galvanized steel pipe column was 3.0 m (Fig. 1). The insect-proof net used in this study, with a mesh dimension of 0.6 mm × 0.6 mm, was made of round polyethylene monofilaments. The diameter of the wire was 0.27 mm and the porosity of the insect-proof net was 0.47. The season-averaged transmissivity of insect-proof net to solar radiation was 70%.

For each treatment, the same amount of fertilizer was applied, including 270 kg N hm^−2^, 67.5 kg P_2_O_5_ hm^−2^, and 135 kg K_2_O hm^−2^. N in the form of urea (*N* = 46%) was applied as follows: 40% as base fertilizer, 20% as tiller fertilizer, 20% as spikelet-promoting fertilizer, and 20% as panicle fertilizer. For the main phosphorous source, compound fertilizer (N: P_2_O_5_: K_2_O = 15%: 15%: 15%) was applied entirely as the basal fertilizer. Potassium chloride (K_2_O = 60%) was applied as the base fertilizer and spikelet-promoting fertilizer at equal amounts. In addition, 3,750 kg hm^−2^ of pig manure (total nutrient content of N, P_2_O_5_, and K_2_O >7%) was applied to the experimental plots. Biological and chemical pesticide was applied to control rice pest insects and diseases during the whole rice-growing period for treatments under OFC, while no pesticide was used for the treatments under IPNC. The application time, type and dose of pesticides are shown in Table 1. All other cultivation practices were carried out according to the local standard of rice cultivation.

**Table 1:** The application time, type and dose of pesticides under open filed cultivation.

Time of application	Type of pesticides	Dose of pesticides (kg hm^−2^)
Seedling	Chlorantraniliprole	0.030
	Buprofezin	0.113
	Kasugamycin	0.024
Tillering	Hexaconazole	0.068
	Pymetrozine	0.084
Booting	Hexaconazole	0.068
Heading	Pymetrozine	0.084
	Kasugamycin	0.024
	Propiconazole	0.110

### Measurements and analysis

#### Micrometeorological data

Two portable automatic weather stations (HOBO U30/NRC; Onset Computer Corp., Bourne, MA, USA) were used to record the micrometeorological data (Air temperature, relative humidity, soil temperature, air speed, gust speed, and solar radiation) every 10 s from transplanting stage to maturity (from June 22 to October 23 in 2011 and from June 19 to October 25 in 2012). One weather station was positioned inside the screenhouse at the center point. The other was placed in ambient conditions. Each weather station contained a suite of meteorological instrumentation that was mounted to the metal t-posts. Solar radiation and photosynthetically active radiation (PAR) inside and outside the screenhouse were measured using a solar radiation sensor (S-LIB-M003, Onset Computer Corp.) and a PAR sensor (S-LIA-M003; Onset Computer Corp.), which were placed at the height of 1.7 m above the soil surface. An air speed sensor (S-WCA-M003; Onset Computer Corp.) was attached at a height of 1.8 m, and a temperature and relative humidity sensor (S-THB-M002; Onset Computer Corp.) was attached at a height of 1.7 m above the soil surface. A solar radiation value of 1.0 W m^−2^ was applied as the day/night threshold to determine daytime and nighttime averages for the meteorological parameters (Perillo et al.,  2015).

#### Plant sampling

Ten hills per plot of rice were selected and labeled to count the tiller number at the main growth stages. Three hills per plot of rice with three replications (nine hills in total) were randomly sampled at transplanting, effective tiller critical leaf stage, jointing, heading, mid-ripening, and maturity, respectively. The samples were separated into leaves, stems (including leaf sheath), and panicles. All plant parts were dried at 105 °C for 30 min, and then at 80 °C until a constant weight was achieved to determine the dry matter accumulation. The leaf area was measured using a LAI-3000 Portable Area Meter (LI-COR, Lincoln, NE, USA). At maturity, ten hills per plot of rice were randomly selected to determine the grain yield and yield components, such as panicle number, spikelet number per panicle, seed-setting rate, and 1,000-grain weight.

#### Tillering traits

The dynamics of tillering (from emergence to the time when the maximum tiller number occurred) could be fitted over days after emergence using a logistic equation, Eq. (1) (Sparkes, Holme & Gaju, 2006; Xie, Mayes & Sparkes, 2015).

(1) \begin{eqnarray*}& & TN= \frac{A}{1+{e}^{-B(t-M)}} \end{eqnarray*}

where *TN* was the tiller number; *A* was the maximum tiller number; *B* was the initial tillering rate; *M* was the days after emergence at which the *TN* was 50% of *A*, and *t* was the days after emergence. Tiller survival, time of tillering onset, time of tillering cessation, tillering duration, and tillering rate were calculated as follows based on the parameters of Eq. (1).

• Tiller survival = panicle number/*A*.
• Time of tillering onset (*t*_*to*_) = *M* − 2.1972∕*B*.
• Time of tillering cessation (*t*_*tc*_) = *M* + 2.1972∕*B*.
• Tillering duration (*t*_*td*_) = *t*_*tc*_ − *t*_*to*_.
• Tillering rate = 0.8*A*/*t*_*td*_.

#### Leaf area duration (LAD) and mean net assimilation rate (mNAR)

After examining several possible functions for goodness of fit, the rational equation, Eq. (2), was used to describe the relationship between leaf area index (LAI) and days after emergence. The function describing LAI, Eq. (2), was integrated from *i* days after emergence (*t*_*i*_) to *j* days after emergence (*t*_*j*_) to generate an estimate of leaf area duration (*LAD*_*i*−*j*_) (Eq. 3). The mean net assimilation rate from *t*_*i*_ to *t*_*j*_ (*mNAR*_*i*−*j*_) was calculated as the quotient of *DMA*_*i*−*j*_ and *LAD*_*i*−*j*_ (Eq. 4) (Andrewh, Matt & Robertp, 2009).

(2) \begin{eqnarray*}& & LAI= \frac{a+bt}{1+ct+d{t}^{2}} \end{eqnarray*}

(3) \begin{eqnarray*}& & LA{D}_{i-j}=\int \nolimits \nolimits _{i}^{j}{f}_{LAI}(t)dt\end{eqnarray*}

(4) \begin{eqnarray*}& & mNA{R}_{i-j}= \frac{DM{A}_{i-j}}{LA{D}_{i-j}} .\end{eqnarray*}

*DMA*_*i*−*j*_, *LAD*_*i*−*j*_, and *mNAR*_*i*−*j*_, were the dry matter accumulation, leaf area duration, and mean net assimilation rate during the growth phase from *i* days after emergence to *j* days after emergence, respectively.

### Statistical analysis

Statistical analyses were carried out using SPSS statistical software (SPSS 20.0). Two years of experimental data were analyzed using analysis of variance (ANOVA) to evaluate the effects of cultivation environment on microclimatic parameters and the effects of year, cultivar, cultivation environment, and their interactions on the leaf area index, dry matter accumulation, leaf area duration, mean net assimilation rate, and grain yield. The least significant difference (LSD) test was used to determine the significance of differences between means at the 0.05 level. Multi-linear regression (MLR) was used to quantify the contribution of yield components to grain yield and the contribution of leaf area duration and net assimilation rate to dry matter accumulation.

## Results

### Effects of insect-proof nets cultivation on microclimatic parameters

**Figure 2:**
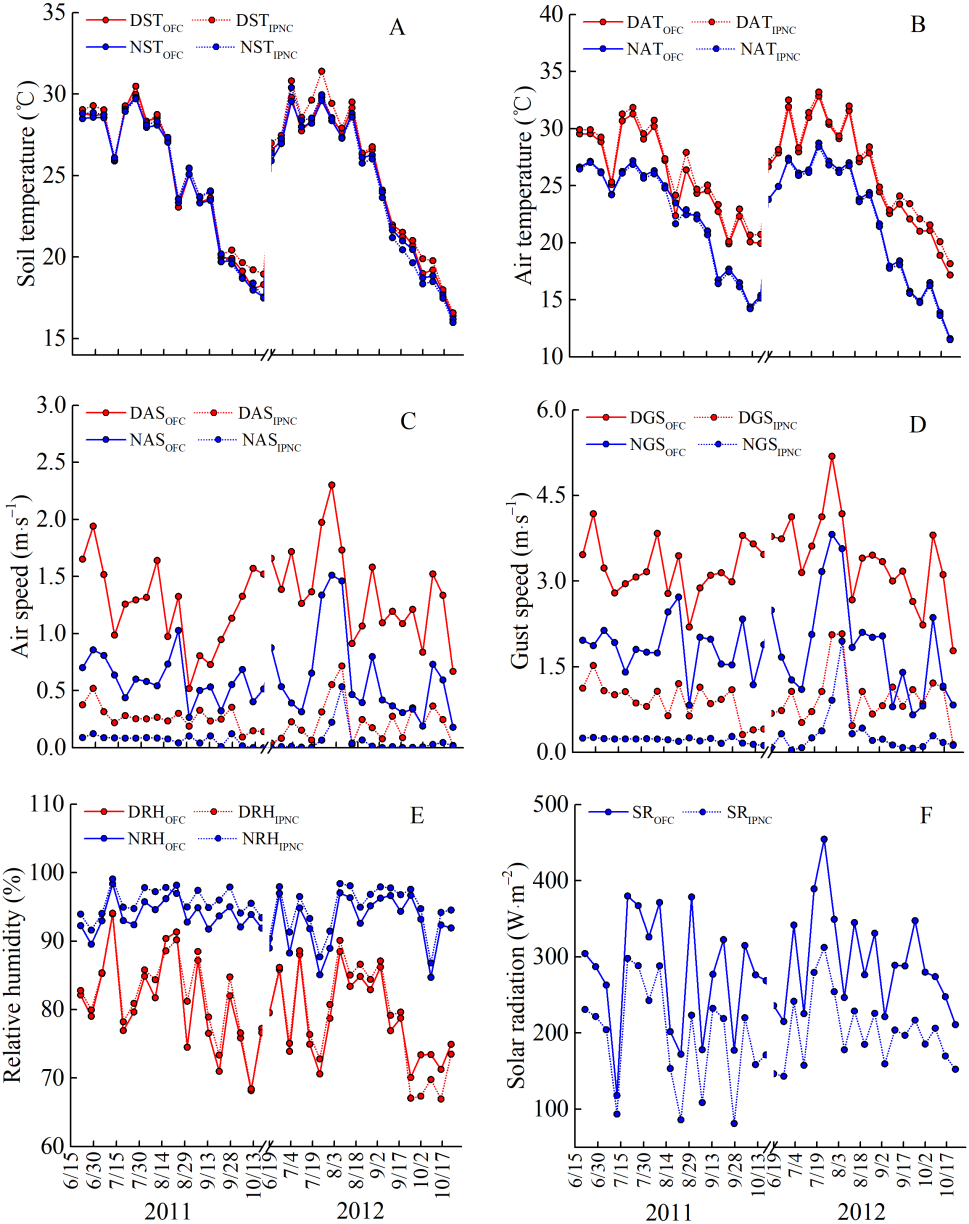
Meteorological parameters under open field cultivation (OFC) and insect-proof nets cultivation (IPNC) in 2011 and 2012 rice growing seasons. DST and NST, daytime soil temperature and nighttime soil temperature (A); DAT and NAT, daytime air temperature and nighttime air temperature (B); DAS and NAS, daytime air speed and nighttime air speed (C); DGS and NGS, daytime gust speed and nighttime gust speed (D); DRH and NRH, daytime relative humidity and nighttime relative humidity (E); SR, solar radiation (F).

The microclimatic parameters in two rice growing seasons were quite different (Fig. 2). The air speed and gust speed were markedly faster in 2012 than in 2011, while the relative humidity was lower in 2011 than in 2012. In 2012, the daytime soil and air temperature were relatively higher, while the nighttime soil and air temperature were relatively lower, than those in 2012. Additionally, the solar radiation was markedly higher than that in 2011 (Fig. 2).

**Figure 3:**
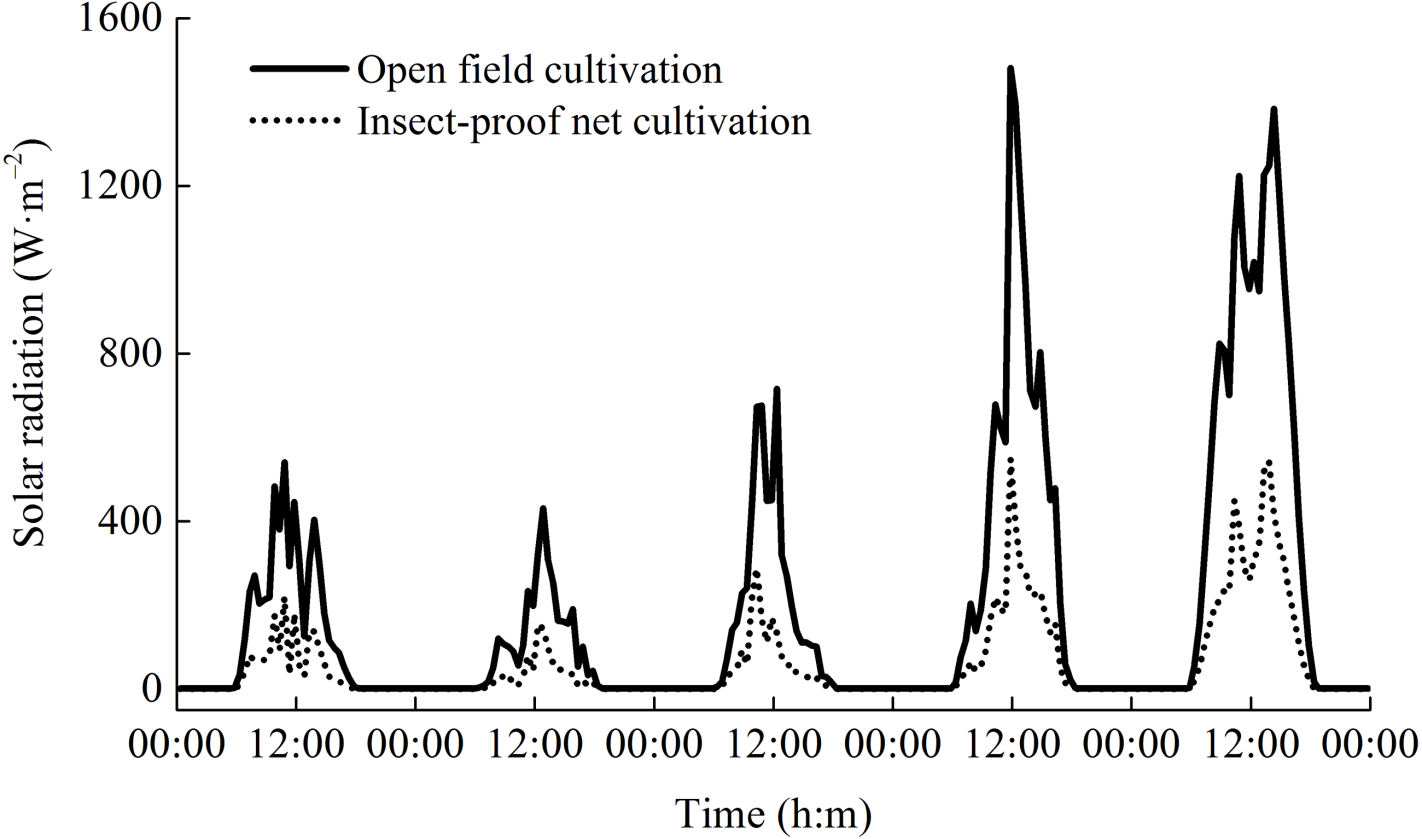
The dynamics of daily solar radiation under open field cultivation and insect-proof nets cultivation (Data were obtained from Sep 9 to Sep 14, 2011).

During the two rice growing seasons, the differences in meteorological parameters between two cultivation environments were obvious (Fig. 2). Daytime soil temperature (1.3–1.9%), daytime air temperature (2.3–2.5%), and nighttime relative humidity (2.0%) under IPNC were slightly higher than under OFC, while the nighttime soil temperature, nighttime air temperature, and daytime relative humidity were relatively unaffected by IPNC during the rice growing seasons. Compared with OFC, IPNC significantly decreased the daytime air speed and nighttime air speed, by 78.6–83.5% (−0.98 m s^−1^ to −1.16 m s^−1^) and 88.2–90.4% (−0.53 m s^−1^ to −0.58 m s^−1^) during the rice growing seasons, respectively. Similarly, IPNC significantly decreased the daytime and nighttime gust speed during the rice growing season by 71.2–71.8% (−2.31 m s^−1^ to −2.46 m s^−1^) and 82.6–88.0% (−1.56 m s^−1^ to −1.61 m s^−1^) during the rice growing season, respectively. In addition, the solar radiation under IPNC was 29.3–31.0% lower than those under OFC (Fig. 2). The extent of solar radiation reduction depended on the time of day and sky conditions. The maximum daily difference in solar radiation received between IPNC and OFC was observed at noon when the highest solar radiation occurred (Fig. 3).

**Table 2:** Effect of insect-proof nets cultivation on grain yield and yield components of Suxiangjing3 (SXJ3) and Nanjing44 (NJ44) in 2011 and 2012.

Year	Cultivar	Cultivation environment	Panicle number (10^4^ hm^−2^)	Spikelet number per panicle	1,000-grain weight (g)	Seed-setting rate (%)	Grain yield (kg hm^−2^)
2011	SXJ3	OFC	399.16^b^	104.87^d^	20.46^c^	82.14^b^	7,037.60^c^
		IPNC	350.44^c^	98.37^e^	19.41^d^	77.41^de^	5,180.19^e^
	NJ44	OFC	253.86^e^	146.95^a^	27.41^a^	84.72^a^	8,189.24^b^
		IPNC	223.94^f^	135.05^b^	26.15^b^	81.58^bc^	6,448.23^d^
2012	SXJ3	OFC	481.58^a^	94.39^ef^	20.25^c^	79.53^cd^	7,321.11^c^
		IPNC	407.43^b^	90.86^f^	19.34^d^	75.64^e^	5,376.69^e^
	NJ44	OFC	288.65^d^	137.84^b^	27.34^a^	83.50^ab^	9,084.82^a^
		IPNC	258.46^e^	125.62^c^	26.17^b^	82.11^b^	6,976.92^c^
ANOVA results		GS	^**^	^**^	ns	ns	^**^
		C	^**^	^**^	^**^	^**^	^**^
		CE	^**^	^**^	^**^	^**^	^**^
		GS*C	^**^	ns	ns	ns	^*^
		GS*CE	^*^	ns	ns	ns	ns
		GS*CE	^**^	^*^	ns	ns	ns
		GS*C*CE	^*^	ns	ns	ns	ns

**Notes.**

Within the same column, means followed by the same letters are not significantly different at 0.05 level.

* *p* < 0.05.

** *p* < 0.01.

ns not significant OFC open field cultivation IPNC insect-proof nets cultivation C cultivar CE cultivation environment

### Effects of IPNC on grain yield and yield components of different rice cultivars

Grain yield was significantly higher in 2012 than in 2011, and significantly higher for Nanjing44 (NJ44) than for Suxiangjing3 (SXJ3) in both years (*p* < 0.01). Compared with OFC, IPNC significantly decreased the grain yield of the two rice cultivars (*p* < 0.01), and the negative effects of IPNC on grain yield were larger for SXJ3 than for NJ44 in both years. In addition, the interaction effects of the growing season and the cultivation environment on grain yield were not significant, suggesting a similar effect of IPNC on grain yield in both growing seasons. For the yield components, IPNC significantly decreased the panicle number, spikelet number per panicle, 1,000-grain weight, and seed-setting rate of the two rice cultivars in both years (*p* < 0.01) for SXJ3 and NJ44 (Table 2). Multiple linear regression analysis showed that the panicle number had a more significant contribution to grain yield (standardized regression coefficient (SRC) = 0.75, *p* < 0.01 for SXJ3 and SRC = 0.76, *p* < 0.01 for NJ44) than the spikelet number per panicle (SRC = 0.40, *p* < 0.01 for SXJ3 and SRC = 0.42, *p* < 0.01 for NJ44), 1,000-grain weight (SRC = 0.19, *p* > 0.05 for SXJ3 and SRC = 0.13, *p* > 0.05 for NJ44), and seed-setting rate (SRC = 0.15, *p* > 0.05 for SXJ3 and SRC = 0.002, *p* > 0.05 for NJ44). This indicated that the grain yield reductions of the two rice cultivars under IPNC were mainly explained by the decreased panicle number.

The effects of IPNC on tillering traits are shown in Table 3. The tillering traits were significantly affected by growing seasons and cultivars (*p* < 0.01), except for the effects of cultivars on tillering duration and initial tillering rate (*p* > 0.05). The maximum tiller number, time of tillering onset, time of tillering cessation, and tillering rate were significantly higher in 2012 than in 2011 and were significantly higher for SXJ3 than for NJ44 (*p* < 0.01). Meanwhile tiller survival was significantly lower in 2012 than in 2011, and was significantly lower for SXJ3 than for NJ44 (*p* < 0.01). The effects of IPNC on tillering traits were significant (*p* < 0.01), except for its effect on time of tillering onset. Under IPNC, the maximum tiller number, tiller survival, initial tillering rate, and tillering rate decreased significantly, on average by 7.7, 6.6, 10.4, and 17.3%, respectively, for SXJ3, and by 7.1, 4.3, 15.0, and 21.1%, respectively, for NJ44. The time of tillering cessation and tillering duration increased significantly, on average by 4.6 and 13.4%, respectively, for SXJ3, and by 7.0 and 18.6%, respectively, for NJ44. Furthermore, the interaction effects of growing season and cultivation environment on the tillering traits were not significant, suggesting a similar effect of IPNC on tillering traits in both growing seasons. However, the interaction effects of cultivar and cultivation environment on the maximum tiller number, initial tillering rate, and tillering rate were significant (*p* < 0.01) (Table 3).

**Table 3:** Effect of insect-proof nets cultivation on tillering traits of Suxiangjing3 (SXJ3) and Nanjing44 (NJ44) in 2011 and 2012.

Year	Cultivar	Cultivation environment	Maximum tiller number (10^4^ hm^−2^)	Tiller survival	Time of tillering onset (d)	Time of tillering Cessation (d)	Tillering duration (d)	Initial tillering rate (10^4^ hm^−2^ d^−1^)	Tillering rate (10^4^ hm^−2^ d^−1^)
2011	SXJ3	OFC	502.26^c^	0.80^bc^	27^b^	52^de^	25^c^	0.17^b^	15.82^b^
		IPNC	460.39^d^	0.76^d^	26^b^	54^c^	28^b^	0.16^cd^	13.19^c^
	NJ44	OFC	298.65^g^	0.85^a^	24^c^	47^f^	23^d^	0.19^a^	10.20^e^
		IPNC	275.33^h^	0.81^b^	22^d^	50^e^	28^ab^	0.16^d^	7.80^g^
2012	SXJ3	OFC	618.93^a^	0.78^cd^	31^a^	56^ab^	25^c^	0.17^b^	19.67^a^
		IPNC	575.69^b^	0.71^e^	31^a^	59^a^	29^ab^	0.15^de^	16.12^b^
	NJ44	OFC	355.77^e^	0.81^b^	27^b^	53^cd^	26^c^	0.17^bc^	10.99^d^
		IPNC	332.70^f^	0.78^cd^	27^b^	57^b^	30^a^	0.15^e^	8.94^f^
ANOVA results		GS	^**^	^**^	^**^	^**^	^**^	^**^	^**^
		C	^**^	^**^	^**^	^**^	ns	ns	^**^
		CE	^**^	^**^	ns	^**^	^**^	^**^	^**^
		GS*C	^**^	ns	ns	ns	^*^	^**^	^**^
		GS*CE	ns	ns	ns	ns	ns	ns	ns
		GS*CE	^**^	ns	ns	ns	ns	^**^	^**^
		GS*C*CE	ns	ns	ns	ns	ns	^*^	^**^

**Notes.**

Within the same column, means followed by the same letters are not significantly different at 0.05 level.

* *p* < 0.05.

** *p* < 0.01.

ns not significant OFC open field cultivation IPNC insectproof nets cultivation C cultivar CE cultivation environment

As shown in Table 4, the panicle number was mainly dependent on the maximum tiller number rather than on tiller survival under IPNC. The maximum tiller number correlated highly positively with the time of tillering onset and the tillering rate (*p* < 0.01), and correlated weakly positively with tillering cessation (*p* < 0.05) for both rice cultivars under IPNC, indicating that the later the time of tillering onset and tillering cessation, and the faster tillering rate, the higher the maximum tiller number. Tiller survival was significantly positively associated with the initial tillering rate (*p* < 0.01), while it was significantly negatively correlated with time of tillering cessation and tillering duration for both rice cultivars under IPNC (*p* < 0.01) (Table 4).

**Table 4:** Correlations between tillering traits of Suxiangjing3 (SXJ3) and Nanjing44 (NJ44) under insect-proof nets cultivation.

Cultivar	Tillering trait	Tiller survival	Maximum tiller number	Time of tillering onset	Time of tillering cessation	Tillering duration	Initial tillering rate	Tillering rate
SXJ3	Panicle number	0.17	0.92^**^	0.78^**^	0.30	−0.58^*^	0.45	0.99^**^
	Tiller survival	–	−0.23	−0.48	−0.88^**^	−0.89^**^	0.91^**^	0.17
	Maximum tiller number	–	–	0.96^**^	0.64^*^	−0.23	0.09	0.92^**^
NJ44	Panicle number	−0.06	0.94^**^	0.83^**^	0.34	−0.28	0.19	0.89^**^
	Tiller survival	–	−0.40	−0.52	−0.92^**^	−0.89^**^	0.90^**^	0.33
	Maximum tiller number	–	–	0.95^**^	0.64^*^	0.06	−0.15	0.70^**^

**Notes.**

* *p* < 0.05.

** *p* < 0.01.

ns not significant

### Effects of IPNC on leaf area index of different rice cultivars

**Figure 4:**
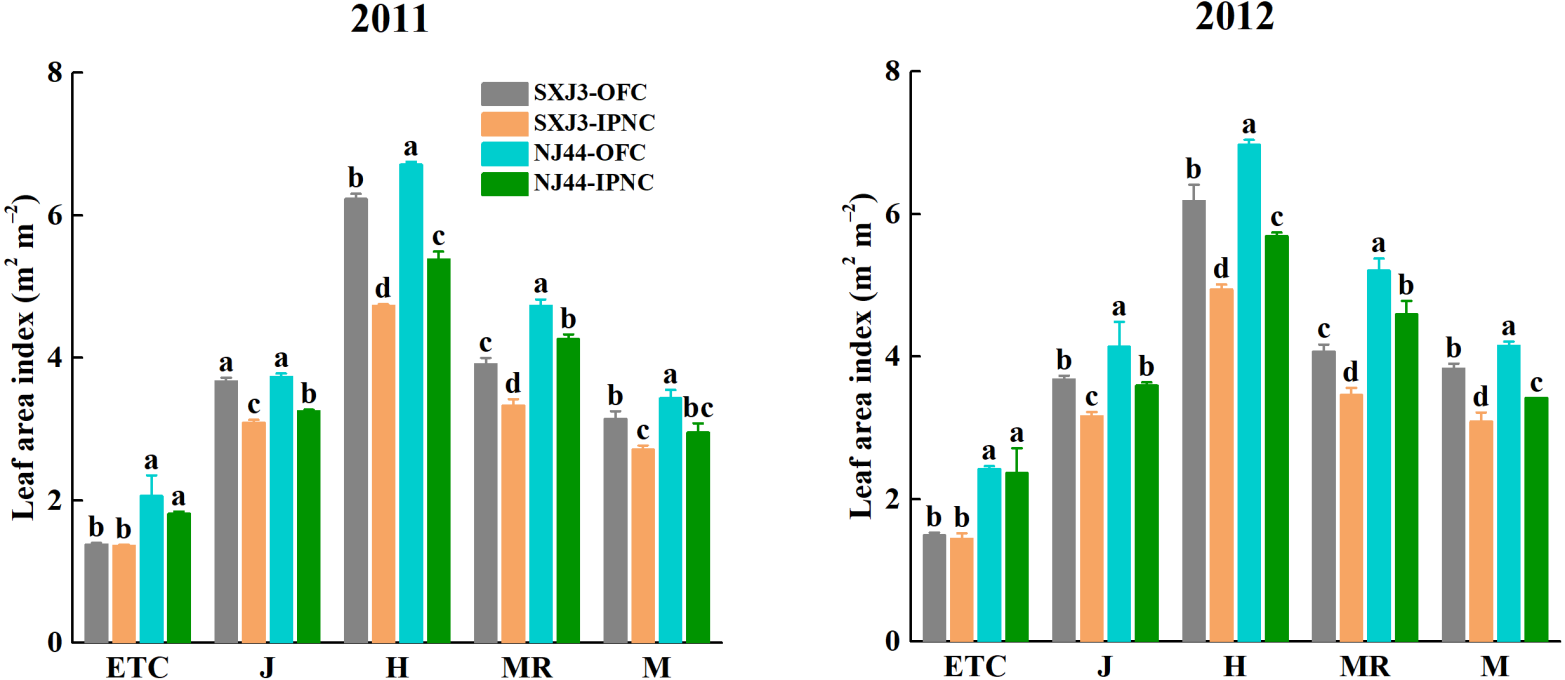
Effects of insect-proof nets cultivation on leaf area index of Suxiangjing3 (SXJ3) and Nanjing44 (NJ44) at different stages in 2011 and 2012. OFC, open field cultivation; IPNC, insect-proof nets cultivation; ETC, effective tiller critical leaf stage; J, jointing; H, heading; MR, mid-ripening; M, maturity. Different lowercase letters indicate significant differences at 0.05 level. Vertical bars represent standard deviation of mean (*n* = 3, standard deviation of three replications) .

The leaf area index (LAI) was higher in 2012 than in 2011 and was higher for NJ44 than for SXJ3 in both years. IPNC significantly decreased LAI of two rice cultivars from jointing to maturity in both years (*p* < 0.05). The LAI reductions of NJ44 from jointing to maturity were lower than those of SXJ3. Compared with OFC, LAI under IPNC decreased on average by 14.9% at jointing, by 20.1% at heading, by 14.9% at mid-ripening, and by 16.6% at maturity for SXJ3, while in NJ44 the average reductions were 13.0% at jointing, 19.1% at heading, 11.0% at mid-ripening, and 15.8% at maturity (Fig. 4).

**Figure 5:**
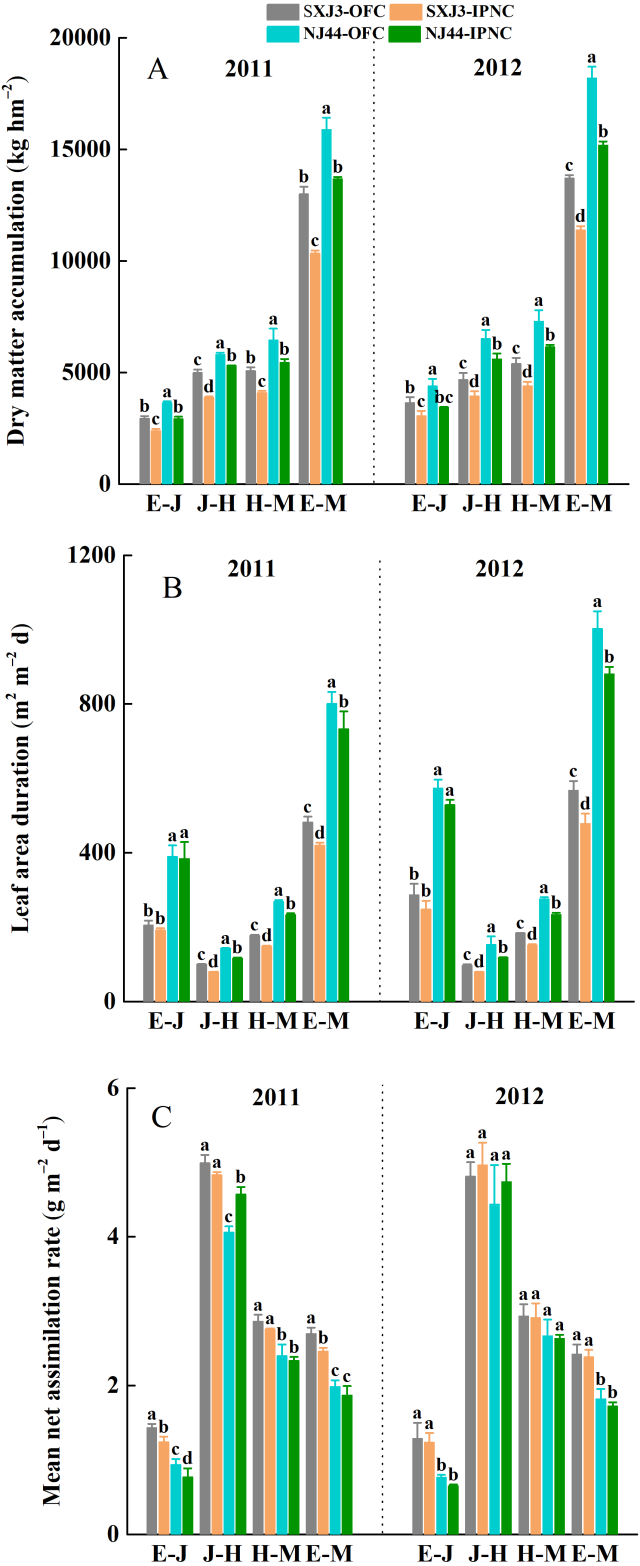
Effects of insect-proof nets cultivation on dry matter accumulation (DMA) (A), leaf area duration (LAD) (B) and mean net assimilation rate (mNAR) (C) during different growth phases of Suxiangjing3 (SXJ3) and Nanjing44 (NJ44) in 2011 and 2012. OFC, open field cultivation; IPNC, insect-proof nets cultivation; E–J, from emergence to jointing; J–H, from jointing to heading; H–M, from heading to maturity; E–M, from emergence to maturity. Different lowercase letters indicate significant differences at 0.05 level. Vertical bars represent standard deviation of mean (*n* = 3, standard deviation of three replications).

### Effects of IPNC on dry matter production of different rice cultivars

The total dry matter accumulation (DMA) was significantly higher in 2012 than in 2011, and significantly higher for NJ44 than for SXJ3 in both years (*p* < 0.05). IPNC significantly decreased the DMA at different growth phases (*p* < 0.05), and the DMA reductions of SXJ3 were larger than those of NJ44 under IPNC, from jointing to maturity. Compared with OFC, IPNC decreased the DMA of SXJ3 and NJ44 on average by 19.0 and 10.9% from jointing to heading, by 19.0 and 16.0% from heading to maturity, and by 17.2 and 16.7% from emergence to maturity, respectively (Fig. 5A). In line with the DMA reduction, a significant negative effect of IPNC was detected on LAD from jointing to heading, heading to maturity, and emergence to maturity for both rice cultivars (*p* < 0.05). When the data were averaged over two years, the LAD of SXJ3 and NJ44 decreased on average by 18.6 and 17.0% from jointing to heading, by 17.3 and 14.5% from heading to maturity, and by 16.2 and 14.4% from emergence to maturity, respectively (Fig. 5B). The effect of IPNC on mNAR of both rice cultivars from emergence to maturity over two growing seasons was not significant, except for the effect of IPNC on mNAR of SXJ3 in 2011. Further analysis on mNAR during different growth phases showed that the mNAR of the two rice cultivars from emergence to jointing decreased significantly under IPNC in 2011 (*p* < 0.05), while the mNAR of both rice cultivars from emergence to jointing only slightly decreased in 2012. The effects of IPNC on mNAR after jointing were insignificant in both years, except for the effect of IPNC on mNAR of NJ44 in 2011 (Fig. 5C). Multiple linear regression analysis was used to evaluate the relationship between LAD, mNAR, and DMA. The standardized regression coefficient and contribution rate showed that both LAD and mNAR had a significant contribution to DMA for both rice cultivars; however, LAD had a greater contribution to DMA than did mNAR (Table 5).

## Discussion

In the present study, the dry matter accumulation and grain yield were significant higher in 2012 than in 2011. The daytime air temperature and solar radiation in 2012 were higher than those in 2011, while the nighttime air temperature was relatively lower than that in 2011. The higher air temperature and solar radiation in the daytime in 2012 than in 2011 could have increased accumulation of assimilates (Islam & Morison, 1992; Dong et al., 2011). Meanwhile, the cooler air temperature at night in 2012 tended to decrease nighttime respiration, which accounted for lower carbon losses (Mohammed & Tarpley, 2009; Laza et al., 2015). These factors resulted in higher dry matter accumulation and grain yield in 2012 than in 2011.

Insect-proof nets significantly alter the microclimate inside the screenhouse, thus creating a different microenvironment compared with ambient conditions (Tanny, 2013). Previous studies suggested that insect-proof nets increased resistance to air flow and thus reduced the ventilation, which may cause temperature increases (Möller et al., 2003; Tanny, 2013). In the present study, the air temperature increased slightly inside the screenhouse. This was consistent with several previous studies that reported minimal changes in air temperature between nets and open field conditions (Shahak, 2008; Collins, Weldon & Taylor, 2010; Kitta et al., 2014; Perillo et al., 2015). The soil temperature also increased slightly inside the screenhouse, which was similar to previous studies that reported that the average soil temperature inside the screenhouse was slightly higher than that outside (Collins, Weldon & Taylor, 2010; Guo et al., 2015). Previous studies reported that humidity was higher in insect-proof nets than in open field conditions, caused by reduced ventilation and decreased removal of water vapor from the nets (Kittas et al., 2006). In the present study, IPNC slightly increased the nighttime relative humidity, while daytime relative humidity was relatively unaffected.

**Table 5:** The contribution of leaf area duration and mean net assimilation rate to dry matter accumulation during different growth phases of Suxiangjing3 (SXJ3) and Nanjing44 (NJ44) under insect-proof nets cultivation.

Cultivar	Growth phase	Leaf area duration		Mean net assimilation rate	
		SRC	CR	SRC	CR
SXJ3	Emergence to jointing	1.07^**^	0.84	0.67^**^	0.15
	Jointing to heading	0.93^**^	0.89	0.30^**^	0.11
	Heading to maturity	0.84^**^	0.78	0.38^**^	0.22
	Emergence to maturity	1.00^**^	0.85	0.54^**^	0.15
NJ44	Emergence to jointing	1.26^**^	0.86	0.93^**^	0.13
	Jointing to heading	1.70^**^	0.99	1.36^**^	−0.02
	Heading to g maturity	0.67^**^	0.53	0.62^**^	0.47
	Emergence to maturity	1.16^**^	0.96	0.66^**^	0.04

**Notes.**

* *p* < 0.05.

** *p* < 0.01.

ns not significant

SRC and CR denote standardized regression coefficient and contribution rate, respectively.

A major effect of insect-proof nets is to increase the resistance to airflow, and thus decrease the internal air speed (Kittas et al., 2006). In the present study, 81.1 and 89.3% reductions in daytime and nighttime air speed, respectively, were observed inside the screenhouse. Our results were similar with the findings of Desmarais, Ratti & Raghavan (1999) who reported an 80% reduction in air speed inside a 3.2 m height screenhouse compared with that in OFC. However, Allen (1975) observed a 67% reduction in air speed in a covered soybean field as compared to an open soybean field. Tanny, Liu & Cohen (2006) reported a 40% reduction in air speed at 5 m height inside a banana screenhouse, in which screen height and plant height were 6 m and 4.2 m, respectively. Siqueira, Katul & Tanny (2012) reported that air speed increased with height from the canopy top to the horizontal screen and decreased by 30% at 5 m height inside the screenhouse compared with that in OFC. The internal air speed is mainly affected by screen type, mesh size, vegetation height, and measurement height (Tanny, 2013). A previous study showed that mean air speed was 18% higher under a knitted screen than under a woven screen (Pirkner et al., 2014). In addition, the mean air speed inside the screenhouse decreased with decreasing mesh size (Harmanto Tantau & Salokhe, 2006) and decreasing measurement height (Siqueira, Katul & Tanny, 2012). These might be the main reasons for the differences in air speed between our study and certain previous studies. For example, the non-dimensional velocity measurement height in our study was an average 0.30, which was similar to the value reported by Allen (1975). However, the hole size of the insect-proof nets used in our study was about 0.60 mm × 0.60 mm, which were smaller than the screens used by Allen (1975) (hole size 2.1 mm × 2.1 mm), which may partially explain the lower air speed observed in our study.

Solar radiation was the microclimatic parameter most affected by the insect-proof nets (Lawson et al., 1994; Kitta et al., 2014). A previous study reported that insect-proof nets significantly reduced solar radiation, and the extent of solar radiation reduction depended on net properties and the solar elevation angle (Tanny, 2013). Our results showed that IPNC significantly reduced solar radiation received by the rice plants, particularly at noon when the maximum solar radiation occurred. Our results were similar to the findings reported by Perillo et al. (2015). Although the global solar radiation decreased under IPNC, IPNC modified the light regime by increasing the ratio of diffuse to beam radiation, which had a positive effect on plant growth (Kitta et al., 2014).

Previous studies reported that net covering significantly increased crop yield in semiarid areas because they mitigated the negative effects of environmental stresses on plants (Kitta et al., 2014). Meanwhile, a net covering could increase the relative fraction of diffuse radiation inside the screenhouse, causing increased radiation use efficiency and photosynthesis (Kittas et al., 2012; Tanny, 2013). However, Liu et al. (2017) reported that rice plants experienced a marked yield reduction under insect-proof nets because of the reduction in solar radiation. In the present study, compared with OFC, IPNC decreased the rice yield on average by 26.5% for SXJ3 and 22.2% for NJ44. This indicated that the positive effect of net covering could not offset the negative effect caused by solar radiation reduction on rice yield. Moreover, the present study showed that the extent of yield reduction induced by decreased solar radiation inside the screenhouse was larger in SXJ3 than in NJ44. Our results were consistent with previous studies, which reported that the extent of yield reduction caused by shading differed in different genotypes (Mu et al., 2010; Li et al., 2010; Pan et al., 2016). In addition, our present study showed that yield reduction under IPNC mainly resulted from decreased panicle number, which was consistent with the findings of Liu et al. (2017).

Tiller production and survival determine the final panicle number and play key roles in grain formation in cereal crop yield (Xie, Mayes & Sparkes, 2015). In the present study, both the maximum tiller number and tiller survival significantly decreased under IPNC in both rice cultivars. Further analysis showed that the decreased maximum tiller number could be largely attributed to reduced tillering rather than the prolonged tillering duration, which accorded with the results of Xie, Mayes & Sparkes (2015). Low solar radiation due to net covering inhibited tillering and enhanced tiller mortality, which probably resulted from an assimilate shortage for tiller buds or developing tillers (Xie, Mayes & Sparkes, 2015). By contrast, light quality, especially the red/far-red ratio, had an independent effect on tillering (Evers, Vos & Andrieu, 2006). Insect-proof nets decreased the red/far-red, which reduced the tillering rate (Casal, 1988; Xie, Mayes & Sparkes, 2015), thereby decreasing the maximum tiller number. In addition, genotypic variation in the response of tillering production to IPNC was observed in the present study. The reduction of panicle number, maximum tiller number, and tiller survival for SXJ3 caused by net covering was larger than that for NJ44, which indicated that NJ44 had a stable tillering capacity in comparison with SXJ3 under IPNC.

In the present study, the leaf area index (LAI) significantly decreased under IPNC, possibly caused by the lower solar radiation inside the screenhouse (Mu et al., 2010). This was consistent with the findings of Perillo et al. (2015), who reported that the LAI of soybean was 20% lower inside the nets, which was mainly attributed to the lower solar radiation (shading intensities of 42%). By contrast, Li et al. (2010) found that shading (shading intensities of 8, 15, and 23%) from jointing to maturity increased the LAI at both 10 and 30 days after anthesis. The effects of shading were determined not only by the shading intensity, but also by the crop growth stage where shading occurred. Cai & Luo (1999) investigated the effects of 45% shading at different growth stages on LAI of rice. They found that shading during the early growth phase caused a significant reduction in LAI compared with the control because of low leaf number per plant and total leaf areas. However, shading during the middle growth phase increased the LAI to a certain degree, and the effect of shading during the later growth phase on the LAI was not significant. In the present study, solar radiation was lower under insect-proof nets than in the open field conditions during the whole growth phase of rice. Shading because of net covering, especially during the early growth phase, affected the leaf expansion rate, leading to a significant reduction in final leaf areas (Moore et al., 2003; Perillo et al., 2015). The reduced LAI decreased light interception and the photosynthetic capacity, leading to reduced dry matter production (Kottmann, Wilde & Schittenhelm, 2016).

Reduced air speed and a changed radiation regime because of net covering greatly affected the total dry matter production (Perillo et al., 2015). Lower air speed inside the screenhouse increased the heat load on the leaves (Norman & Campbell, 1998), thereby potentially decreasing the rate of photosynthesis. The screenhouse significantly reduced the ventilation rate, which reduced the supply of CO_2_ from outside (Harmanto Tantau & Salokhe, 2006; Tanny, 2013). Meanwhile, photosynthesis by rice plants consumed a large amount of CO_2_ inside the screenhouse, finally leading to an insufficient supply of CO_2_ for photosynthesis. Shading caused by net covering was another factor that greatly affected the dry matter accumulation. Perillo et al. (2015) reported that the total dry matter accumulation of soybean increased by 30% under 42% shading in insect-proof cages compared with OFC. Kittas et al. (2012) found that the total dry matter accumulation of tomato grown under the nets with shading intensities of 34, 40, and 49% were significantly higher than those grown under OFC. Kitta et al. (2014) also found that sweet pepper productivity significantly increased under three covering materials with different shading intensities (a pearl insect-proof net with a shading intensity of 22%, a white insect proof net with a shading intensity of 41%, and a green shade-net with a shading intensity of 38%). Li et al. (2010) suggested that the total dry matter accumulation increased significantly under the 5 and 15% shading, while 22% shading induced a significant reduction in the total dry matter accumulation. This indicated that the threshold value of shading intensity that induces a significant reduction in crop productivity varies in different crop genotypes. In the present study, compared with OFC, the dry matter accumulation at maturity of SXJ3 and NJ44 decreased on average by 18.6 and 15.2%, respectively, under shading of 30% inside the screenhouse. The results of our study were similar to the results reported by Mu et al. (2010), who reported that crop productivity experienced a significant decrease under 22 and 33% shading treatments in wheat. Dry matter accumulation is a product of the LAD and the mNAR. Our results showed that the LAD decreased on average by 14.3% for SXJ3 and 10.3% for NJ44 under IPNC, which mostly resulted from the decreased LAI. In contrast to the LAD, the mNAR only slightly decreased under IPNC (−5.1% for SXJ3 and −5.5% for NJ44). This indicated that mNAR was less sensitive to IPNC than LAD. This was because the higher leaf stomata conductance and leaf nitrogen might alleviate the decrease in photosynthetic rate of the leaves near the top of the canopy inside the screenhouse compared with those in OFC. In addition, the photosynthetic rate of the shaded leaves near the bottom of the canopy may have increased because of the increase in the average diffuse and scattered light intensity inside the screenhouse compared with those under ambient conditions (Perillo et al., 2015). Therefore, these positive effects of net covering greatly alleviated the decrease in the overall net assimilation rate of the canopy under the low light intensity inside the screenhouse.

## Conclusion

This study revealed that insect-proof nets altered the microclimate inside the screenhouse in comparison with open field conditions, mainly by reducing the air speed and changing the radiation regime, which ultimately negatively affected dry matter production and yield formation. Yield losses under insect-proof nets were mainly caused by the decreased panicle number, which was attributed to the reduced maximum tiller number. The maximum tiller number was largely dependent on the tillering rate rather than tillering duration. Additionally, reduced leaf area duration caused by the reduced leaf area index was the major reason for decreased dry matter accumulation under insect-proof nets, while the mean net assimilation rate was relatively unaffected by the insect-proof nets. The responses to insect-proof nets in this study are critical for providing a theoretical basis to improve the agronomic performance of pesticide-free rice under insect-proof nets cultivation for agronomy researchers and field management farmers. It should be noted that the sun angle, intensity of solar radiation, and extreme environmental conditions that occurred during the experiments will likely affect the magnitude of the influence of insect-proof nets on the experimental results (Perillo et al., 2015). Thus, more validation is needed under a wider range of environment conditions in the near future.

##  Supplemental Information

10.7717/peerj.6135/supp-1 Data S1 Raw data

## Additional Information and Declarations

### Data Availability

The following information was supplied regarding data availability: Raw data is provided in the Supplemental Files.
